# Atomic scale DFT based investigation of tuning and quantum modulation of zinc blende ZnS bandgap for next-generation materials

**DOI:** 10.1039/d5ra02719e

**Published:** 2025-07-17

**Authors:** Suneela Arif

**Affiliations:** a Department of Physics, Hazara University (HU) Mansehra Pakistan suneela@hu.edu.pk (092) 310-050-7841

## Abstract

This study reveals the electric-field-induced tuning and modulation of electronic bandgap of zinc blende ZnS as potential prospect for the next generation optoelectronics. By employing Generalized Gradient Approximation (GGA) with a Plane-Wave basis set based on the Quantum espresso package, the correlation between electronic bandgap engineering, tuning/switchable modulations with the varying applied electric field strength is established. We unveiled dynamical switching in range from 2.37 eV to 0 eV (at critical field) under the positive field strength of 0.01 V Å^−1^ to 0.5 V Å^−1^, and from 2.41 eV to 1.52 eV under the negative fields strength from −0.01 V Å^−1^ to −0.5 V Å^−1^ along the out-of-plane *z*-axis. The valence and conduction bands overlap at a critical field (0.5 V Å^−1^) is attributed due to the Mott transition, where electron–electron interactions persuade a transition in behavior from semiconductor to conductor. The partial (PDOS) and total density of states (TDOS) display electric-field-tailored dynamically switching of the sp^3^ hybridization into the Zn-3d, Zn-2s and S-2p states. The modulation of local density of states (LDOS), charge density and variation in charge transfer (between Zn and S) further confirm electric-field-induced redistribution of charges between Zn and S atoms. Optical parameters, comprising refractive index (*n*(*ω*)), absorption coefficient (*α*(*ω*)), reflectivity (*R*(*ω*)), extinction co-efficient (*k*(*ω*)), real (*ε*_1_(*ω*)) and imaginary (*ε*_2_*ω*)) dielectric function and electron energy loss (ELS)) display field-dependent behavior, signifying the potential of ZnS as a tunable optoelectronic material. These findings validate the feasibility of electric-field-controlled engineering of ZnS properties, paving the way for exciting advancements in the controlled functionalities in semiconductors to design innovative next-generation optoelectronic and photonic devices.

## Abstract 2

3D visualization of electric-field-induced dynamical modulation in charge density in zinc blende ZnS from 0.01 V Å^−1^ to 0.3 V Å^−1^ and −0.01 V Å^−1^ to −0.3 V Å^−1^.

## Graphical abstract

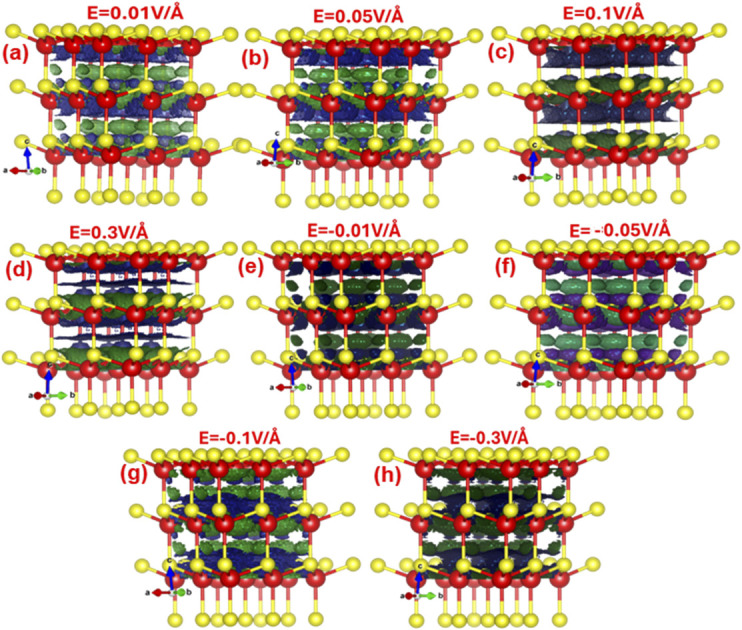

## Introduction

II–VI semiconductors have gained substantial scientific interest due to promising potential in the next generation microelectronic, optoelectronic and spintronic tecnologies.^1–3^ Among these materials, zinc blende phase sphalerite zinc sulfide (*a* = *b* = *c* = 5.4093 Å, *α* = *β* = *γ* = 90°, space group *F*-43*m*, space group # 216 and cell volume is 158.279 Å^3^)^3–5^ have been widely studied due to wide bandgap in the range from 3.68–3.9 eV (analogous wavelength of 337 nm at 300 K), stability and compatibility, high optical (visible light) transparency, good electrical conductivity, optical transmittance, photoluminescence, high exciton binding energy (3 meV) and high refractive index.^6–8^ These fascinating physical properties have opened new avenue as an indispensable part of the solar cell industry,^3,4^ emission/detection and modulation of visible and near ultraviolet region of electromagnetic spectrum,^7,8^ blue-light diodes,^8^ electro-luminescent displays,^9,10^ phosphorescence devices^11^*etc.* ZnS is the subject of scientific interest due to its inherent optoelectronic properties,^3–7^ which make it template for prospective applications in the blue-green region of the electromagnetic spectrum, containing blue lasers and optical waveguides. Zinc blende ZnS has been prepared in various morphologies, including bulk crystal,^12,13^ nanoparticles,^6^ nanorods^14^ and thin film^15^*via* both chemical solution-based methods (*e.g.*, sol–gel, chemical coprecipitation, electrochemical *etc.*)^13–16^ and chemical vapor deposition techniques (*e.g.*, CVD, MOCVD, MOVPE, MBE *etc.*).^15,17–19^ In addition, the potential of these materials has been further tuned and improved through doping various transition metals (*e.g.*, Fe, Ni, Cr, Co), forming dilute magnetic semiconductors.^19–23^ The structural, electronic, optical, magnetic, phase transition and mechanical properties of zinc blende ZnS have also been theoretically studied by executing various computational models and theoretical approximations within the density functional theory (DFT).^22,23^ DFT studies offer insights for understanding atomic-scale physical mechanisms, complementing experimental results by offering a theoretical framework to explain and predict material behavior at the quantum level.^24–27^

Despite the thorough experimental and theoretical work available on ZnS, most of the research work executed in past has ignored the contribution from the Zn-3d orbital states by assuming them as a part of the chemically inert atomic cores may consequence in causing the difficulties in depiction of the physical properties of ZnS more precisely.^28,29^ The role of d-states has recently been investigated through photoemission spectra, which revealed signals from the cation d-bands. In addition, calculations that treat cation d-electrons as valence electrons verify their dynamic influence on the physical properties of II–VI and III–V semiconductor.^27–29^ So, to achieve precise results d states orbitals are considered. Moreover, while extensive research exists on the structural, electronic, optoelectronic, and optical properties mediated by intrinsic stimuli and engineered through mechanical strain, there remains a scarcity of information regarding the effects of extrinsic stimuli, such as the application of an electric field. Therefore, the ability to tune and manipulate these properties *via* electric field as external stimuli will present new avenue of tailoring the materials properties lead to improving specific applications. Consequently, systematic studies on the potential to tune and modify zinc blende ZnS using external electric fields as a stimulus are highly desirable to meet the growing demands of advanced miniaturized microelectronic industries.

Therefore, utilizing first-principles calculations within the framework of density functional theory (DFT),^25–29,34,35^ this paper 1st ever reports the electric field controlled optoelectronic response of zinc blende phase ZnS.^30^ Using systematic DFT calculations^24–27^ with a Plane-Wave basis set and pseudopotentials (PP) through the generalized gradient approximation (GGA) implemented in Quantum Espresso,^31–33^ we aim to elucidate the mechanisms by which the electric field alters, engineer, and modulate the electronic structure, bandgap, charge carrier dynamics, and optoelectronic properties in a controlled fashion. Based on the theoretical predictions, we accomplish that this research delivers valuable insights, introducing a groundbreaking concept of stimuli-responsive control over functionalities lead to valuable prospect for the next-generation miniaturized microelectronic, optoelectronic, and photoluminescence devices.

## Computational details

The computational calculations for the zinc blende phase sphalerite ZnS were executed by means of Quantum Espresso simulation package^31–33^ within framework of density functional theory (DFT)^24–27^ using plane wave basis set.^31–33^ The generalized gradient approximation (GGA)^33–35^ was conducted systematically step-by-step with and without applied electric (−0.01 V Å^−1^ → −0.5 V Å^−1^, 0.0 V Å^−1^ and 0.01 V Å^−1^ → 0.5 V Å^−1^ along the out-of-plane *z*-axis) to perform structural relaxation and to calculate electronic bandgap engineering, as well as to monitor the controlled modulations and dynamics in the total (TDOS) and partial densities of states (PDOS). The exchange correlation potential was calculated by means of Perdew–Burke–Ernerhof (PBE) non-linear core correction functional.^31–35^ The energy cutoff for the plane wave expansion was set at 40 Ry, and a *k*-point grid of 8 × 8 × 8 was used in the first Brillouin zone. The convergence criteria for both self-consistent (SCF) and non-self-consistent (NSCF) calculations^35,36^ was set within 10^−5^ eV. Turbo Time-Dependent Density Functional Theory (TTDFT) method^37^ was used to calculate the electron energy loss spectrum (EELS) and real (*ε*_r_(*ω*)) and imaginary (*ε*_i_(*ω*)) dielectric function. Furthermore, the values of real (*ε*_r_(*ω*)) part of the dielectric function were extracted using the Kramers–Kronig relationship,^38,39^ where the values of imaginary (*ε*_i_(*ω*)) part of dielectric function were obtained by summing the transitions from occupied valence states to unoccupied conduction states. The optical parameters, such as refractive indices (*n*(*ω*)), optical reflectivity (*R*(*ω*)), extinction coefficient (*k*(*ω*)), absorption coefficient and optical conductivity (*σ*(*ω*)) of the zinc blende ZnS is calculated with GGA approximation.^33–35^

## Results and discussion

**Fig. 1:**
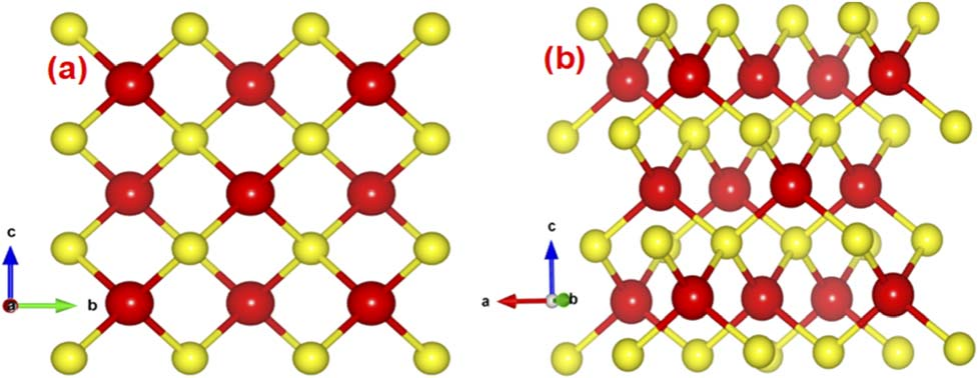
Crystal structure of zinc blende ZnS (a) 2D view (b) 3D View.

To accomplish systematic computation precisely, we accomplished a series of calculations for the unit cell optimization conclude equilibrium lattice constant *a* = *b* = *c* = 5.41 Å, which is in good agreement with the previously reported experimental results.^6–9,13–22^ Under standard temperature and pressure (STP), ZnS was found to be in a stable zinc blende (ZB or B_3_) phase, which features a face-centered cubic structure with an internal parameter of (¼, ¼, ¼), as illustrated in Fig. 1(a and b).

Both zinc and sulfur atoms in ZnS are tetrahedrally coordinated, following an ABCABC stacking sequence that exhibits sp^3^ hybridization. The 4s and 4p orbitals of zinc hybridize to form four sp^3^ hybrid orbitals, while each sulfur ion similarly forms four sp^3^ hybrid orbitals through the combination of one 3s orbital and three 3p orbitals, resulting in tetrahedral coordination. The sp^3^ hybrid orbitals from both the zinc cations and sulfur anions overlap to form sigma bonds, reinforcing the tetrahedral structure.

**Fig. 2:**
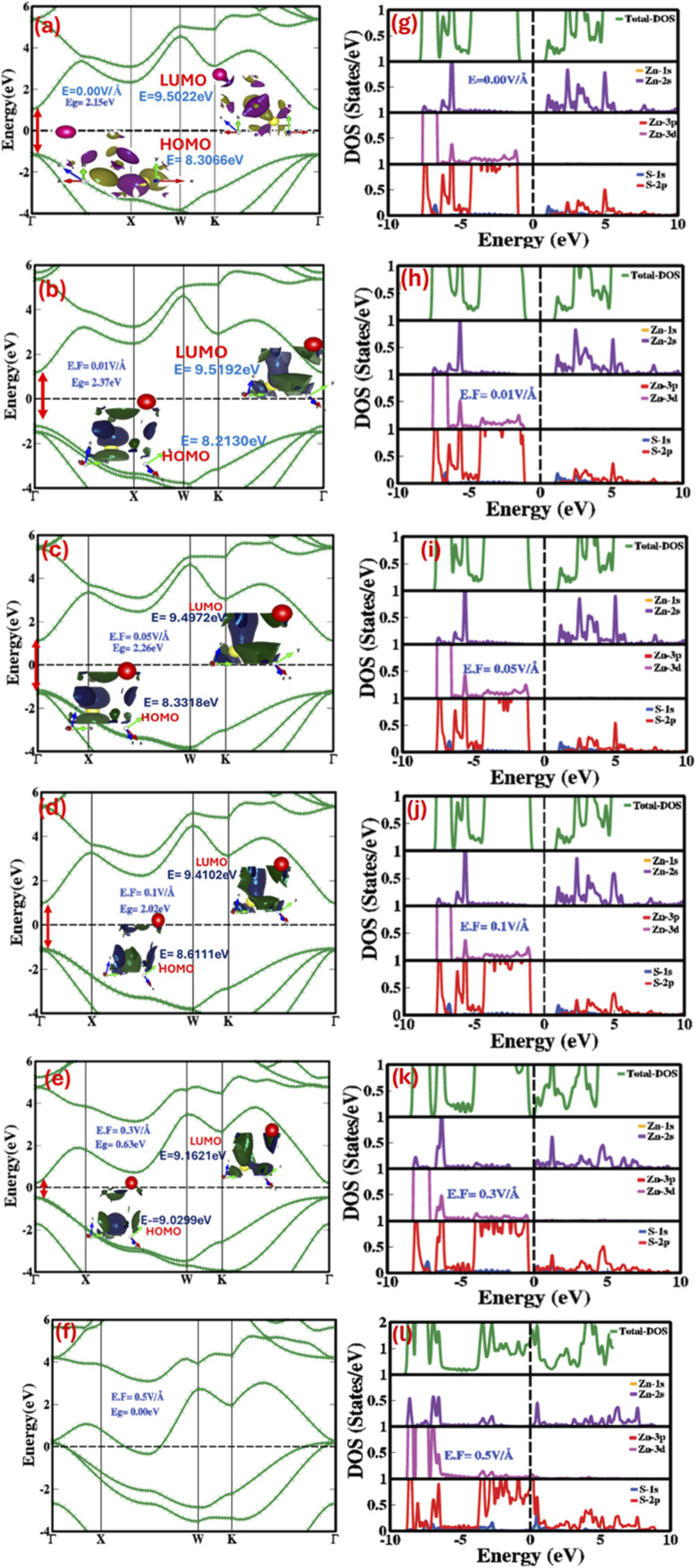
Electric field modulated and engineered (a–f) electronic bandgap (a–e (inset)) HOMO–LUMO energy levels (g–l) partial and total density of states at 0.00 V Å^−1^, 0.01 V Å^−1^, 0.05 V Å^−1^, 0.1 V Å^−1^, 0.3 V Å^−1^ and 0.5 V Å^−1^.

The electronic bandgap offers insights into the electronic properties such as conductor, insulator, semiconductor *etc.* of the materials illustrated by the energy gap between the valence (closely spaced normal energy) bands and conduction (higher energy) bands. Fig. 2(a–l) represents electric field engineered electronic band structure, total density of states (TDOS) and partial density of states (PDOS) with and without externally applied electric field for the zinc blende phase ZnS. We witness that the electronic states in zinc blende ZnS respond to stimuli such as electric fields, potentially enable the advanced photonics and optoelectronic technologies with precise control.

As shown in Fig. 2(a), in the absence of an externally applied electric field, both the valence band maximum (VBM) and conduction band minimum (CBM) are located at the Γ-Γ symmetry point of the Brillouin zone (BZ), confirming that ZnS is a direct wide bandgap material with a bandgap of 2.15 eV at 0.0 V Å^−1^. The highest occupied molecular orbital (HOMO) (inset Fig. 2(a)) is positioned below the Fermi level and is primarily composed of S-2p and Zn-3d orbitals, while the lowest unoccupied molecular orbital (LUMO) arises from Zn-sp^3^ hybrid orbitals. The calculated HOMO–LUMO energy gap for ZnS, without an external electric field, is 2.15 eV. Total (TDOS) and partial density of states (PDOS), shown in Fig. 2(g), imply that in the absence of an external electric field, the conduction bands are predominantly influenced by Zn (2s, 2p) and S (2p) orbitals in the zinc blende ZnS system, with these states primarily occurring in the 4.0 eV to 10 eV energy range. In this energy range substantial overlap between Zn-2s and S-2p orbitals is obvious, revealing both covalent and ionic bonding interactions among the first nearest-neighbor Zn atoms in the zinc blende ZnS structure. The valence bands are mainly composed of two parts, top valence bands (4.9 eV to 0 eV) and bottom valence bands (6.8 eV to 4.9 eV), as illustrated in Fig. 2(g). It is obvious that the top valence bands and bottom valence bands are mainly contributed by S (2p) and Zn (3d) orbits, respectively. In addition, the Zn and S show sp^3^ hybridization at 4.3 eV between the FNN Zn–S. We observed that the electronic bandgap changes systematically with the applied electric field. As the field increases incrementally from 0.01 V Å^−1^ to 0.3 V Å^−1^, the bandgap first increases to 2.37 eV at 0.01 V Å^−1^. However, as the field strength continues to rise, the bandgap decreases, reaching 2.32 eV at 0.03 V Å^−1^, 2.26 eV at 0.05 V Å^−1^, 2.19 eV at 0.07 V Å^−1^, 2.09 eV at 0.09 V Å^−1^, and 2.02 eV at 0.1 V Å^−1^. At 0.3 V Å^−1^, the bandgap drops further to 0.63 eV. Finally, at the critical field of 0.5 V Å^−1^, the valence and conduction bands overlap with the Fermi level, leading to a transition from a semiconductor to a metallic phase. This dynamic modulation of the electronic bandgap, controlled switching, and engineering are governed by the Stark effect, which explains the response of holes in the valence band and electrons in the conduction band to the applied electric field. Applying an external electric field to ZnS interacts with the crystal's dipole moment, causing changes in the potential energy of electrons and holes, and shifting the energy of the conduction band minimum (CBM) and valence band maximum (VBM). The detailed values of electric-field engineered valance band, conduction band and electronic bandgap are listed in Table 1 and represented in Fig. 2(a–f). We witness that increase in the electric field from 0.01 V Å^−1^ to 0.05 V Å^−1^ the bandgap decreases from 2.37 eV to 2.26 eV, such decrease with increasing electric field is due to shift of CBM to lower and VBM shift higher energy states. Further increase in the electric field from 0.07 V Å^−1^ to 0.3 V Å^−1^ lad to converse stark effect due to which CBM goes higher in energy while VBM shift to lower energy states consequences in decrease in electronic bandgap (from 2.19 eV to 0.63 eV). At an applied electric field of 0.5 V Å^−1^ (Fig. 2(f)) the CBM and VBM overlap with Fermi level cause the phase transformation from semiconductor into metallic. Overall, we concluded that applied electric field on the zinc blende ZnS engineers, though the electronic bandgap though the response is nonlinear and higher critical electric field induce substantial changes in valence and conduction bands resulting in phase transformation. The induced bandgap tuning/engineering with electric field can further be validated from the highest occupied molecular orbital (HOMO) and the lowest unoccupied molecular orbitals (LUMO). Fig. 2(a–e) (inset) clearly illustrates that the HOMO–LUMO gap are stimuli response switch and trigger with an applied electric field to 1.2435 eV, 1.1654 eV, 1.0171 eV, 0.8715 eV, 0.7991 eV and 0.1322 eV at 0.01 V Å^−1^, 0.03 V Å^−1^, 0.05 V Å^−1^, 0.07 V Å^−1^, 0.09 V Å^−1^, 0.1 V Å^−1^ and 0.3 V Å^−1^, shows continuous linear decrease in HOMO–LUMO gap with increasing electric field attributed due to interaction of applied electric field with dipole moment of the ZnS crystal. The stimuli responsible change in the HOMO–LUMO gap was further explored by investigating the total (TDOS) and partial density of states (PDOS). Fig. 2(g–l) signify that the valence band and conduction band predominantly comprise of S-2p, Zn-3d, Zn-2s and Zn-2s, Zn-3p, S-2p orbital states. The lowest unoccupied molecular orbital above the Fermi level primarily derives from Zn-sp^3^ hybrid orbitals, while the highest occupied molecular orbital above the Fermi level is formed by S-2p and Zn-3d orbitals. As the electric field increases from 0.01 V Å^−1^ to 0.3 V Å^−1^, the Zn-2s, Zn-3p, and S-2p states in the conduction band shift closer to the Fermi level due to a decrease in their energy, while the valence band states move further away. At 0.5 V Å^−1^, the conduction band overlaps entirely with the Fermi level, causing transition from a semiconductor to a metallic phase. The energy shift in both the valence and conduction band is due to the interaction of the orbitals with the applied electric field. The distribution of densities of states (DOS) as a function of the electric field aligns closely with the variations in bandgap energies and HOMO–LUMO molecular orbital energies.

**Table 1:** Detailed values of electric field induced modulation and tuning of the conduction band (*E*_c_ (eV)), valence band (*E*_v_ (eV)) and electronic bandgap (*E*_g_ (eV) = *E*_c_ (eV)−*E*_v_ (eV)) of zinc blende ZnS

E.F. (V Å^−1^)	*E*_c_ (eV)	*E*_v_ (eV)	*E*_g_ (eV)
0.00	1.01	−1.14	2.15
0.01	1.16	−1.21	2.37
0.02	1.15	−1.20	2.35
0.03	1.14	−1.18	2.32
0.04	1.12	−1.17	2.29
0.05	1.10	−1.16	2.26
0.06	1.08	−1.15	2.23
0.07	1.05	−1.14	2.19
0.08	1.02	−1.14	2.16
0.09	0.99	−1.10	2.09
0.1	0.95	−1.07	2.02
0.2	0.54	−0.71	1.25
0.3	0.17	−0.46	0.63
0.4	0.13	−0.14	0.27
−0.01	1.18	−1.23	2.41
−0.02	1.19	−1.24	2.43
−0.03	1.19	−1.25	2.44
−0.04	1.19	−1.26	2.45
−0.05	1.20	−1.26	2.46
−0.06	1.20	−1.27	2.47
−0.07	1.20	−1.28	2.48
−0.08	1.19	−1.27	2.46
−0.09	1.19	−1.27	2.46
−0.1	1.18	−1.28	2.46
−0.2	1.09	−1.20	2.29
−0.3	0.97	−1.03	2.0
−0.4	0.85	−0.87	1.72
−0.5	0.74	−0.78	1.52

**Fig. 3:**
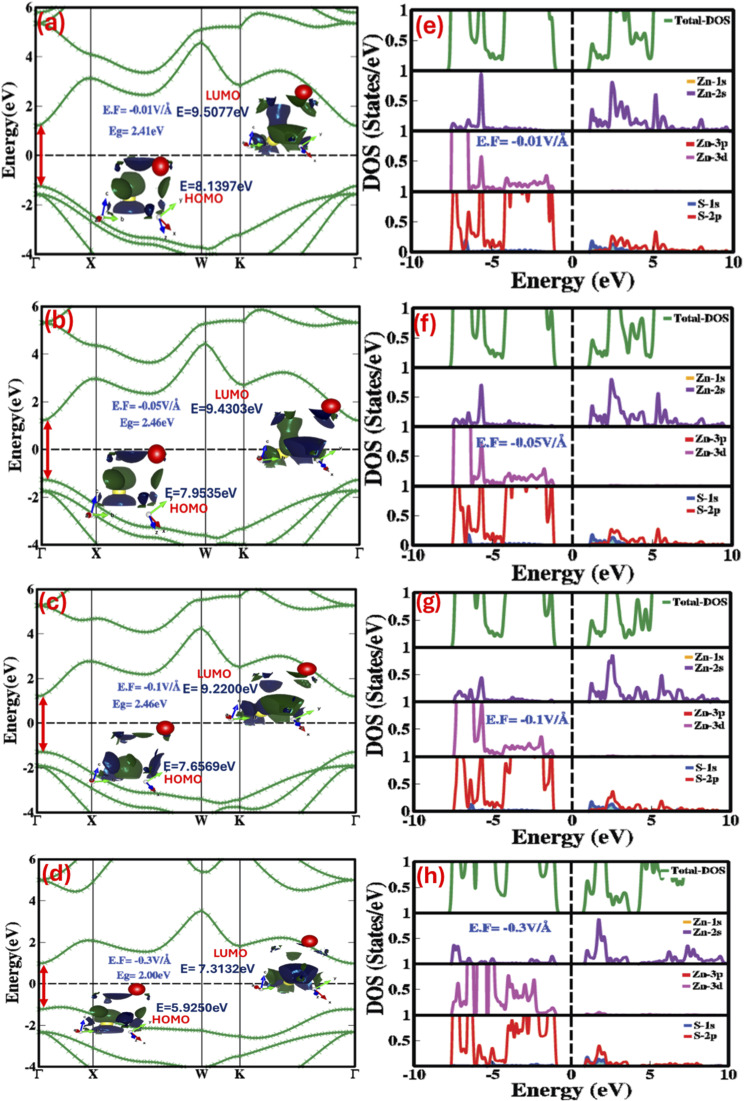
Electric-field-modulated (a–d) electronic bandgap (a–d (inset)) HOMO–LUMO energy levels (e–h) partial (PDOS) and total density of states (TDOS) at −0.01 V Å^−1^, −0.05 V Å^−1^, −0.1 V Å^−1^ and −0.3 V Å^−1^.

To achieve profound insight into the stimuli-responsive behavior, we applied a reverse electric field and examined its influence on the electronic band structure, HOMO–LUMO states, and both partial (PDOS) and total densities of states (TDOS), as shown in Fig. 3. The applied electric field, ranging from −0.01 V Å^−1^ to −0.5 V Å^−1^, induced substantial and non-linear variations in the bandgap, as shown in Fig. 3(a–d), with corresponding values listed in Table 1. Primarily, at −0.01 V Å^−1^, the bandgap surged from 2.15 eV to 2.41 eV. A further rise in the field strength up to −0.07 V Å^−1^ caused a trivial rise to 2.48 eV. Beyond this point, as the field increased from −0.09 V Å^−1^ to −0.5 V Å^−1^, the bandgap reduced remarkably, reaching 1.52 eV.

These findings approve that applying a negative electric field systematically increases the energy levels of both the valence band maximum (VBM) and conduction band minimum (CBM), triggering them to shift away from the Fermi level and ensuing in an increased bandgap. However, in the field range of −0.2 V Å^−1^ to −0.5 V Å^−1^, the energies of the CBM and VBM begin to decrease, shifting toward the Fermi level and thereby reducing the bandgap, as shown in Fig. 3(a–d) and detailed in Table 1. Notably, the effect of a reverse electric field on the HOMO–LUMO gap is the same as a positive electric field. Under negative fields of −0.01 V Å^−1^, −0.03 V Å^−1^, −0.05 V Å^−1^, −0.07 V Å^−1^, −0.09 V Å^−1^, −0.1 V Å^−1^, and −0.3 V Å^−1^, the HOMO–LUMO gap increases almost linearly to 1.368 eV, 1.4257 eV, 1.4768 eV, 1.5189 eV, 1.5509 eV, 1.5631 eV, and 1.3882 eV, respectively. In contrast, the positive electric field induces a decreasing trend in the HOMO–LUMO gap.

The modulation of orbital contributions under an applied electric field can be further interpreted through total density of states (TDOS) and partial density of states (PDOS) analyses. These results reinforce the correlation between electric field strength and variations in the HOMO–LUMO gap and electronic band structure. As shown in Fig. 3(e–h), increasing the negative electric field from −0.01 V Å^−1^ to −0.3 V Å^−1^ predominantly affects the valence band, which is largely governed by Zn-3d, Zn-2s, and S-2p orbitals. In the 1–5 eV range, the valence band is primarily composed of S-2p states, while in the 5–10 eV range, momentous hybridization occurs between Zn-3d and S-2p states. This strong overlap improves the optoelectronic properties of zinc blende ZnS, refining its ability to absorb light and making it a promising candidate for ultraviolet (UV) photodetectors.

Moreover, the effective separation of photogenerated electron–hole pairs is critical for enhancing photocatalytic performance. The Zn-3d and S-2p orbital overlap plays a crucial role in suppressing charge carrier recombination, thereby facilitating improved photocatalytic processes such as pollutant degradation and water splitting.

When we increase the electric field in reverse direction from −0.1 V Å^−1^ to −0.3 V Å^−1^ the S-2p states move from conduction band to valence band thus making valence band entirely dominated by the Zn-3d and S-2p overlapped states. The subsequent overlap of these orbital states develops and improves the optoelectronic and photocatalytic properties.

Table 2 listed the total and partial charges on the individual atoms and the amount of the charge distribution/transfer with the applied varying electric field. It is apparent from data given that when we increase the external electric field from 0.01 V Å^−1^ to 0.5 V Å^−1^, the charge transfer from S atom to Zn atom led to an increase in the total charge on Zn atom from 18.6525 to 18.9090. When we apply electric field in reverse direction from −0.01 V Å^−1^ to −0.5 V Å^−1^ converse is observed and the charge transfer from Zn atom to S atom. According to the data in Table 2, as the electric field increases in the reverse direction from −0.01 V Å^−1^ to −0.3 V Å^−1^, the charge on the Zn atom decreases from 18.6390 to 18.3010, while the charge on the sulfur atom increases from 5.3893 to 5.4236. This overall charge transfer between the Zn and S atoms can be observed through the local density of states (LDOS) and charge density distributions.

**Table 2:** Detailed dynamical variation and controlled modulation in charge transfer between Zn and S atom in zinc blende ZnS with externally applied electric field

E.F. (V Å^−1^)	Zn				S		
	Total	*s*	*p*	*d*	Total	*s*	*p*
0.01	18.6525	2.6822	5.9997	9.9705	7.1804	1.8069	5.3735
0.03	18.6660	2.6953	5.9997	9.9710	7.1614	1.8055	5.3559
0.05	18.6794	2.7086	5.9997	9.9711	7.1405	1.8040	5.3365
0.07	18.6929	2.7223	5.9997	9.9708	7.1177	1.8025	5.3152
0.09	18.7064	2.7363	5.9997	9.9703	7.0929	1.8009	5.2920
0.1	18.7131	2.7435	5.9997	9.9700	7.0797	1.8000	5.2797
0.3	18.8545	2.9066	5.9995	9.9484	6.7175	1.7937	4.9237
0.5	18.9090	3.0078	5.9992	9.9020	5.9504	1.7728	4.1776
−0.01	18.6390	2.6696	5.9997	9.9697	7.1975	1.8082	5.3893
−0.03	18.6256	2.6572	5.9997	9.9686	7.2127	1.8093	5.4034
−0.05	18.6121	2.6453	5.9997	9.9671	7.2260	1.8103	5.4157
−0.07	18.5986	2.6337	5.9997	9.9651	7.2375	1.8112	5.4263
−0.09	18.5851	2.6262	5.9997	9.9628	7.2471	1.8120	5.4351
−0.1	18.5783	2.6172	5.9997	9.9614	7.2512	1.8123	5.4389
−0.3	18.4425	2.5389	5.9996	9.9040	7.2366	1.8129	5.4236
−0.5	18.3010	2.5346	5.9990	9.7673	7.0370	1.8039	5.2331

**Fig. 4:**
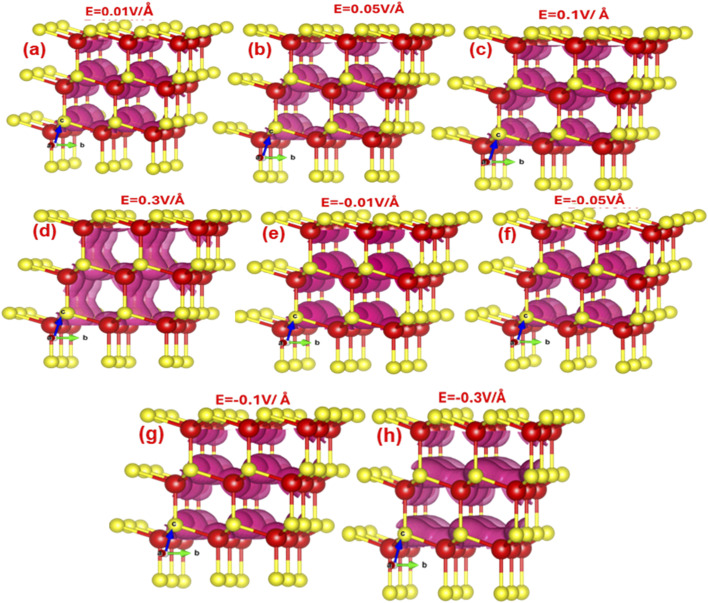
(a–h) 3D visualization of electric-field-modulated local density of states (LDOS with isosurface value of 0.0360951) at 0.01 V Å^−1^, 0.05 V Å^−1^, 0.1 V Å^−1^, 0.3 V Å^−1^, −0.01 V Å^−1^, −0.05 V Å^−1^, −0.1 V Å^−1^, −0.3 V Å^−1^.

Fig. 4(a–h) denotes the electric field engineered and tailored local density of states (LDOS). It is apparent that externally applied electric field mediates band bending, which locally alters the energy distribution of individual states. The LDOS reveals that as the electric field increases from 0.01 V Å^−1^ to 0.09 V Å^−1^, the electron density cloud around the zinc atom (shown in red) happens to be more pronounced compared to that around the sulfur atom. With a further surge in the electric field, the electron cloud on the Zn atom initiates to overlap with the electron density of the S atom.

The increase in electric field from 0.09 V Å^−1^ to 0.3 V Å^−1^, the local density clouds of both Zn and S atoms overlap entirely in the lateral direction leads to local changes in the valence and conduction band edges, triggering an abrupt alteration in the bandgap and transforming ZnS from a semiconductor to a metallic state. Meanwhile, increase in electric field in the reverse direction from −0.01 V Å^−1^ to −0.3 V Å^−1^, triggers the electron density cloud to concentrate more around the sulfur atom rather than the zinc atom.

By −0.3 V Å^−1^, the electron density around both zinc and sulfur atoms overlaps completely in the horizontal direction. The horizontal overlap of Zn–S bond bending causes an increase of the bandgap as listed in Table 1.

**Fig. 5:**
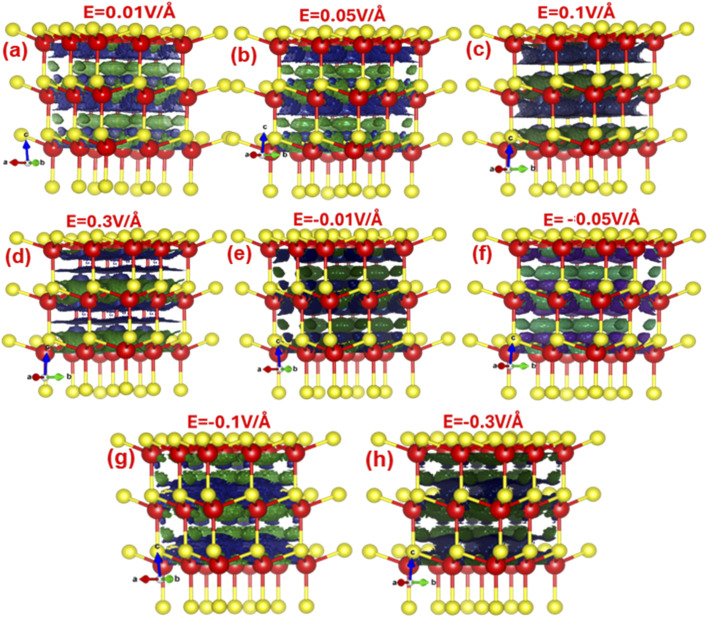
(a–h) 3D visualization of electric-field-induced dynamic modulation and distribution in charge density (at isosurface value of 0.006581) in zinc blende ZnS at 0.01 V Å^−1^, 0.05 V Å^−1^, 0.1 V Å^−1^, 0.3 V Å^−1^, −0.01 V Å^−1^, −0.05 V Å^−1^, −0.1 V Å^−1^, −0.3 V Å^−1^.

The charge density analysis (Fig. 5) reveals that the bond between zinc and sulfur atoms has both covalent and ionic characteristics. Covalent nature emerges from the overlap between the sp hybridized orbitals of the zinc atom and the p orbitals of the sulfur atom. However, the bond also exhibits substantial ionic character due to the transfer of 4s electrons from the zinc atom to the partially filled 3p orbitals of the sulfur atom. In the charge density representation, blue and green colors imply positive and negative values for the difference in charge density, respectively.

Fig. 5 illustrates that as the electric field increases from 0.0 V Å^−1^ to 0.3 V Å^−1^, negative charge accumulates on the zinc atom while a positive charge cloud forms around the sulfur atom, reflecting a transfer of charge from sulfur to zinc. Conversely, when the electric field is increased in the negative direction from −0.01 V Å^−1^ to −0.3 V Å^−1^, negative charge accumulates on the sulfur atom, indicating a transfer of charge from zinc to sulfur. This observation is consistent with the data presented in Table 2.

**Fig. 6:**
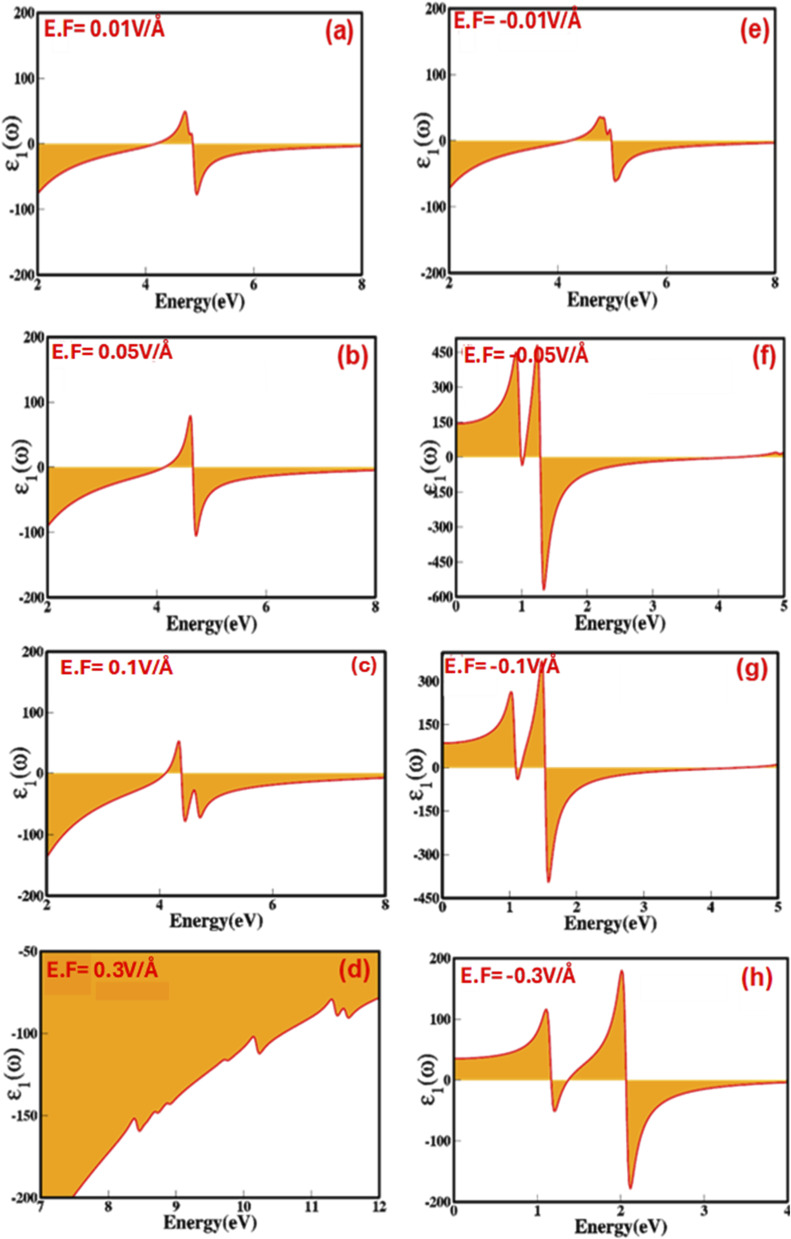
(a–h) Electric-field-induced engineering and modulation of the real part of dielectric function of zinc blende ZnS compounds calculated by GGA process.

**Fig. 7:**
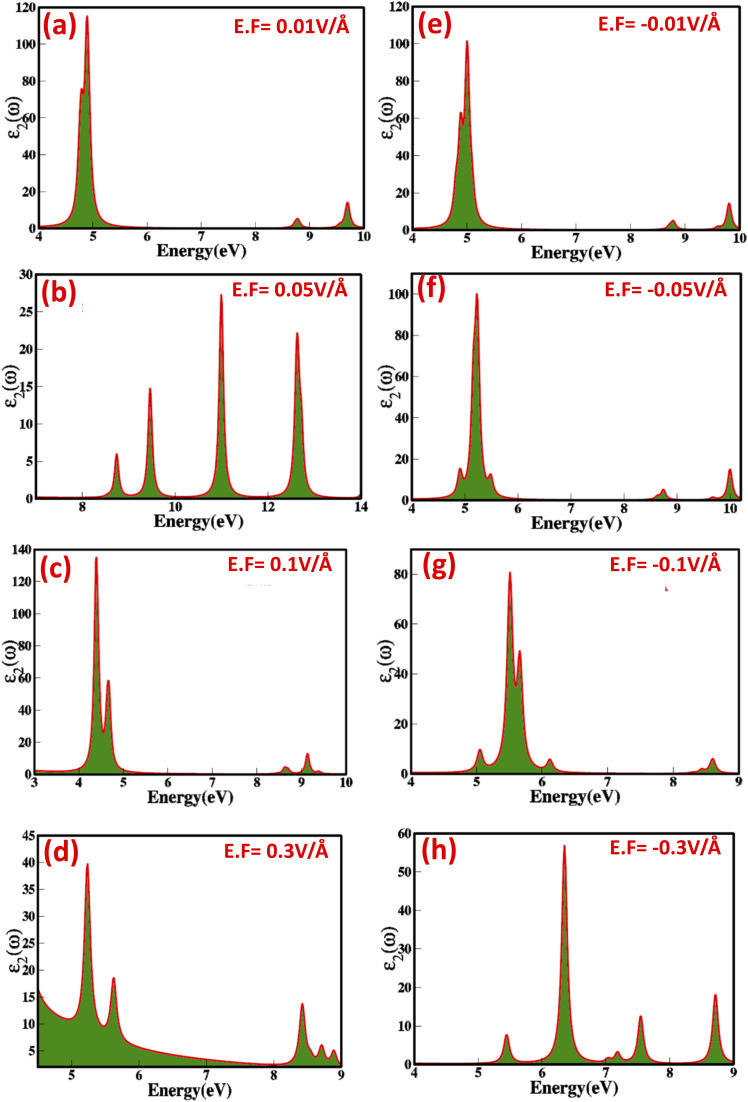
(a–h) Electric-field-induced modulation in the imaginary part of dielectric function of zinc blende ZnS at (a) −0.01 V Å^−1^ (b) −0.05 V Å^−1^ (c) −0.1 V Å^−1^ (d)−0.3 V Å^−1^ (e)−0.01 V Å^−1^ (f) −0.05 V Å^−1^ (g) −0.1 V Å^−1^ (h) −0.3 V Å^−1^.

To explore the prospect of zinc blende ZnS as a stimulus controlled responsive optoelectronic and photonic material, we have analytically examined electric-field-mediated modulation of optical parameters, include, dielectric constant (real (*ε*_1_(*ω*)) and imaginary (*ε*_2_(*ω*)) part of dielectric), refractive index (*n*(*ω*)), optical reflectivity (*R*(*ω*)), extinction co-efficient (*k*(*ω*)) and energy loss function (ELS). The real and imaginary parts of the dielectric function allow us to evaluate and extract some worthy optical parameters. The dynamics and modulations of the real and imaginary dielectric function were calculated under the applied electric field for zinc blende ZnS using the Turbo Time-Dependent Density Functional Theory (TTDFT) method^31–37^*via* Kramers–Kronig relationship^38,39^ and summing the transitions from occupied valence states to unoccupied conduction states. Fig. 6(a–h) and 7(a–h) shows that the real and imaginary part of dielectric constant as a function of the applied external electric field in the range from 0.01 V Å^−1^ to 0.3 V Å^−1^ and −0.01 V Å^−1^ to −0.3 V Å^−1^ prove that zinc blende phase ZnS is electric field switchable and tunable responsive dielectric material.

In Fig. 7(a–h), we observe that as the electric field is increased from 0.00 V Å^−1^ to 0.1 V Å^−1^, the imaginary part of the dielectric constant rises from 18.25 to 52.61. However, at the critical field of 0.3 V Å^−1^, this value unexpectedly drops to 19.54 due to the overlap of valence and conduction states. At 0.0 V Å^−1^, the real part of the dielectric constant is −14.70, changes to −151.88 at 0.3 V Å^−1^ (Fig. 6(a–h) indicates that increasing the electric field causes a shift in energy levels, resulting in reduced polarizability in ZnS because the transition between shifted energy level requires more energy. Average values, real, *ε*_1_ (*ω*), and imaginary, *ε*_2_ (*ω*), part of dielectric constant (considering all visible peaks) under the applied electric field for the zinc blende ZnS as a function of the photon energy are listed in Table 3. The change in values of real part of dielectric constant are inversely correlated with electric field mediated modulations and dynamics in the electronic bandgap (Penn model). Such as, in ZnS the application of external electric field switch and modulate the bandgap which inversely affect real part of dielectric function causes the substantial changes, such as at 0.01 V Å^−1^ the *E*_g_ is 2.37 eV and *ε*_1_ (*ω*) is −14.70, which shifts and at 0.3 V Å^−1^ the *E*_g_ is 0.27 eV and *ε*_1_ (*ω*) is −151.88. Evidence of the dielectric constant being electric field responsive confirms the electric field-controlled nature of the ZnS. The rise in the value of *ε*_1_ (*ω*) from 0.00 V Å^−1^ to 0.1 V Å^−1^ and decrease from −0.01 V Å^−1^ to −0.1 V Å^−1^ agree with the bandgap shift with externally applied electric field. The value of real part dielectric constant at critical points at 0.5 V Å^−1^ confirms the transition of material from semiconductor to conducting mode.

**Table 3:** Electric field modulation and engineered real and imaginary part of dielectric function of zinc blende ZnS

E.F. (V Å^−1^)	Avg. *ε*_2_ (*ω*)	Avg. *ε*_1_ (*ω*)	*ε* (*ω*)	*n* (*ω*)	*k* (*ω*)	*α* (*ω*)	*R* (*ω*)
0.01	18.33	−12.87	5.45	4.36	2.08	26.13	0.47
0.03	52.82	−12.45	53.27	5.11	5.16	64.84	0.68
0.05	46.30	−13.60	32.70	5.56	4.16	52.27	0.63
0.07	17.60	−13.44	4.16	4.21	2.08	26.13	0.46
0.09	45.41	−31.04	14.47	6.56	3.46	43.47	0.62
0.1	51.86	−30.25	25.61	6.84	4.07	51.14	0.64
0.3	52.61	−31.60	21.01	6.81	3.85	48.38	0.64
0.5	19.54	−151.88	−132.34	12.34	0.79	9.92	0.72
−0.01	46.09	−0.51	45.58	4.82	4.77	59.94	0.65
−0.03	46.31	−0.37	45.94	4.83	4.79	60.19	0.66
−0.05	33.45	79.41	112.86	1.83	9.09	114.22	0.91
−0.07	38.08	67.45	105.53	2.23	8.51	106.93	0.89
−0.09	27.30	53.13	86.43	1.81	7.51	94.37	0.88
−0.1	36.18	48.51	84.69	2.45	7.38	92.73	0.85
−0.3	17.38	15.50	32.88	1.97	4.40	55.29	0.72

Fig. 6 and 7 reveal that the absorption peaks for the real and imaginary part of dielectric constant as a function of photon energy (eV) with applied positive (0.01 V Å^−1^ to 0.3 V Å^−1^) and reverse electric field (−0.01 V Å^−1^ to −0.3 V Å^−1^) shift towards the higher and lower energy ranges correspondingly point toward the electric field switchable response. The increased sharpness in the absorption of ZnS confirms a phase transition that aligns with the material's electronic band structure. Overall, the response of the dielectric constant indicates that zinc blende ZnS exhibits absorbance in the UV region.

Our findings on the electric field-tunable dielectric function of zinc blende ZnS recommend that the zinc blende ZnS is appropriate for numerous optoelectronic applications, such as adaptive optics, reconfigurable photonic circuits and where stringent control over the dielectric constant is needed. Electric field induced and modulated optical properties, including optical reflectivity (*R*(*ω*)), absorption coefficient (*α*(*ω*)), refractive index (*n*(*ω*)), and extinction coefficient (*k*(*ω*)) were calculated as functions of photon energy (listed in Table 3). We witnessed that the refractive index features a nonlinear response to the applied electric field exhibit values in the range 5.11, 5.56, 4.21, 6.56, 6.84, and 6.81 at electric fields strength of 0.01 V Å^−1^, 0.03 V Å^−1^, 0.05 V Å^−1^, 0.07 V Å^−1^, 0.09 V Å^−1^, and 0.1 V Å^−1^, respectively. However, the values of refractive index upsurges sharply with an increase in electric field strength to 12.34 at 0.3 V Å^−1^, implying a phase transition from semiconductor to conductor. Similarly, the refractive index values for increasing electric fields in the reverse direction also show a nonlinear response. The extinction coefficient (*k*(*ω*)) specifies the ability of material to absorb per unit distance is calculated using refractive index and the imaginary part of the dielectric function. The detailed values of the electric-field-engineered extinction coefficient are listed in Table 3. It is evident that as the applied electric field increases, the nonlinear optical response of the extinction coefficient (*k*(*ω*)) becomes more pronounced, leading to enhanced multiphoton absorption and, consequently, changes in the extinction coefficient. The interaction of light with ZnS under the applied electric field is illustrated by the absorption coefficient (*α*(*ω*)). The values obtained for the absorption coefficient (shown in Table 3) show inconsistencies and non-linearity in modulation. The optical reflectivity signals the amount of light reflected from the material's surface. Optical reflectivity is derived from refractive index demonstrate nonlinear modulation when subjected to an external electric field (Table 3). The tuning and engineering of optical properties such as refractive index (*n*(*ω*)), extinction coefficient (*k*(*ω*)), absorption coefficient (*α*(*ω*)), and reflectivity (*R*(*ω*)) in response to an external stimulus like an electric field can be described by the Pockels and Kerr effects.

**Fig. 8:**
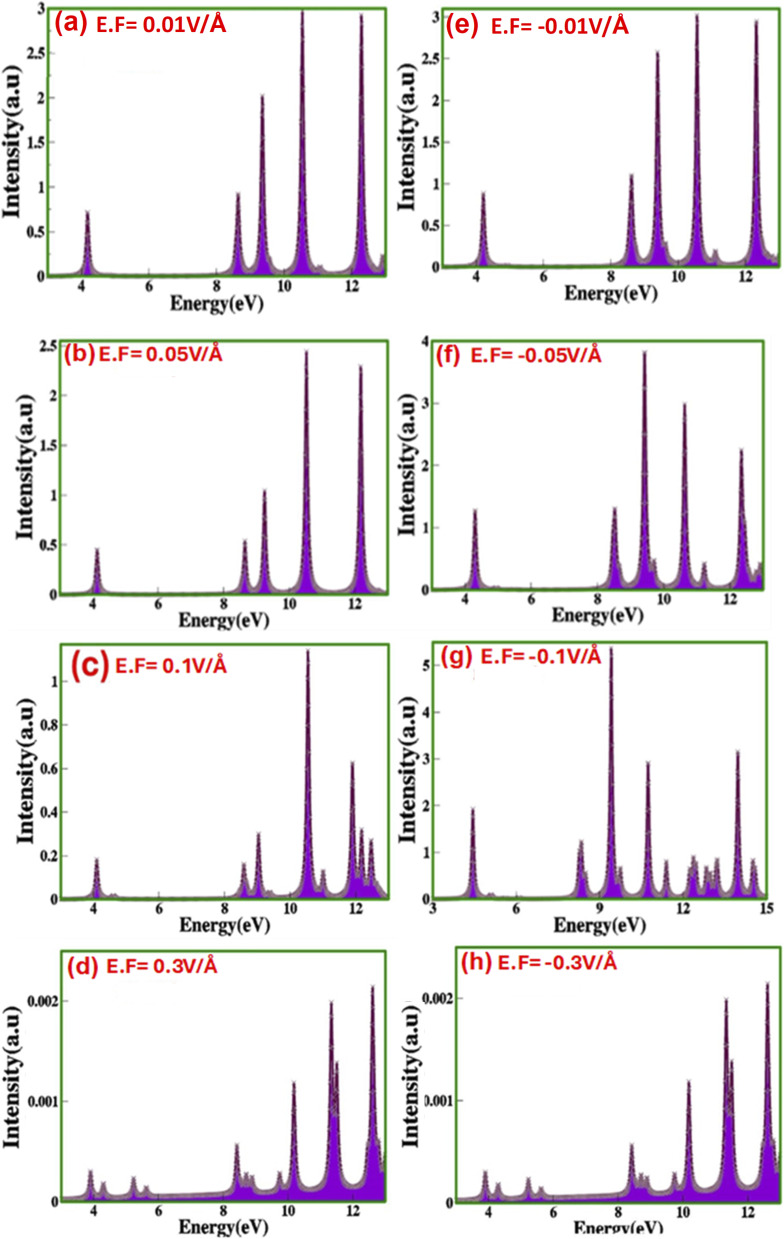
(a–h) Electric-field-induced modulated electron energy loss spectrum (EELS) of zinc blende ZnS under the applied (a) 0.01 V Å^−1^ (b) 0.05 V Å^−1^ (c) 0.1 V Å^−1^ (d) 0.3 V Å^−1^ (e) −0.01 V Å^−1^ (f) −0.05 V Å^−1^ (g) −0.1 V Å^−1^ (h) −0.3 V Å^−1^.

Fig. 8(a–h) represents electric-field-engineered modulation in the electron energy loss spectrum (EELS) of zinc blende ZnS.

We observed that as the electric field increases from 0.01 V Å^−1^ to 0.3 V Å^−1^, numerous peaks begin to appear in the higher energy range of the spectrum. These peaks arise since the incident; photons may lose energy to plasmon excitations several times. As the applied electric field increases from 0.01 V Å^−1^ to 0.3 V Å^−1^, and from −0.01 V Å^−1^ to −0.3 V Å^−1^, electrons gain extra energy. This surge in energy leads to more frequent scattering events, causing additional peaks in the spectrum validating semiconducting nature. However, the transition metals and their oxides commonly exhibit only a single broad plasmon peak, lacking these additional spectral features. The energy loss of an incident electron throughout the scattering can be determined by the dielectric constant, as previously discussed. The peaks observed in the lower and higher energy ranges are attributed to scattering by valence and core electrons, respectively. In the electron energy loss spectrum (EELS), as the electric field increases from −0.01 V Å^−1^ to −0.3 V Å^−1^, the number of peaks decreases. This implies fewer electron scattering events under a negative electric field compared to those witnessed with a positive electric field from 0.01 V Å^−1^ to 0.3 V Å^−1^.

## Conclusions

This study thoroughly reveals the electric-field-induced modulation and tuning of the structural, electronic, and optoelectronic properties of zinc blende ZnS using first-principles density functional theory (DFT) inside the GGA framework *via* the Quantum Espresso platform. Our findings approve that the electronic bandgap, PDOS, TDOS, and crucial optical parameters—such as dielectric function (*ε*_1_(*ω*), *ε*_2_(*ω*)), refractive index (*n*(*ω*)), absorption coefficient (*α*(*ω*)), reflectivity (*R*(*ω*)), extinction coefficient (*k*(*ω*)), and energy loss function (EELS)—can be efficiently engineered and tailored through the application of external electric fields. It is concluded that electronic bandgap systematically decreases with increasing positive electric field (0.01 V Å^−1^ to 0.3 V Å^−1^), and at critical field (0.5 V Å^−1^) transition to metallic behaviour occur. However, converse is the case for negative electric fields (−0.01 V Å^−1^ to −0.3 V Å^−1^) produced the opposite trend. The electric field tunable bandgap in the range from 2.41 eV to 1.52 eV corresponds to a wavelength shift from ∼515 nm to ∼817 nm, covering the visible to near-infrared (NIR) spectrum. This tunability proposes strong prospects for broadband, switchable photodetectors and electro-optic modulators, predominantly in applications such as LiDAR, optical switching, and infrared imaging. A sharp decline in electronic bandgap at 0.3 V Å^−1^ in the range from 2.0 eV to 0.63 eV, near the critical field (0.5 V Å^−1^) point toward strong ON/OFF contrast, which is ideal for high-speed electro-absorption modulators. The systematic dynamic reversibility and non-destructive nature of the electric field-induced modulation also feature prospects in low-power, reconfigurable devices and non-volatile memory technologies. The modulation of electronic structure is correlated to the charge distribution/redistribution between Zn and S atoms, as established by charge density and LDOS analyses. Overlapping between Zn-2s, Zn-3p, and S-2s orbitals concludes strong covalent bonding and electric-field-responsive controlled hybridization. EELS results confirm the shifting of numerous plasmonic peaks due to the scattering of core and valence electrons, further approving the modulation of the electronic structure and optical properties of zinc blende ZnS under an applied electric field strength. Based on our comprehensive and organized investigation of electric-field-controlled modulation and engineering of optoelectronic properties, we accomplish that zinc blende ZnS is a promising candidate for designing and engineering the novel architecture for the next generation smart optoelectronic technology with electric field-controlled dynamics.

## Conflicts of interest

We hereby confirm that the work reported in this manuscript is novel and innovative and has no conflict of interest.

## Data Availability

The raw/processed data required to reproduce these findings cannot be shared at this time as the data also forms part of an ongoing study. Furthermore, the data may be provided on request.
